# Construction of Pickering Double Emulsions Based on Xanthan Gum/Lysozyme Nanoparticles: Structure, Stability, and Co-Encapsulation of Epigallocatechin Gallate and β-Carotene

**DOI:** 10.3390/foods14010098

**Published:** 2025-01-02

**Authors:** Yongpeng Yin, Denglin Luo, Lala Li, Xingguo Li, Mengyao Kang, Bakht Ramin Shah, Xianling Wei, Wei Xu

**Affiliations:** 1 College of Food and Bioengineering, Henan University of Science and Technology, Luoyang 471023, China; yin15939728672@163.com (Y.Y.); kangyao200001@163.com (M.K.); 2 College of Life Science, Xinyang Normal University, Xinyang 464000, China; zhuxianyu2024@163.com; 3 Department of Human Nutrition and Dietetics, Ziauddin University, Karachi 75600, Pakistan; raminshah83@gmail.com; 4 College of Light Industry and Food Science, Zhongkai University of Agriculture and Engineering, Guangzhou 510225, China; xianlingwei@foxmail.com

**Keywords:** pickering double emulsions, emulsion stability, co-encapsulation, epigallocatechin gallate, β-carotene

## Abstract

The low stability of water-in-oil-in-water (W_1_/O/W_2_) double emulsions greatly limits their applications. Therefore, in this study, W_1_/O/W_2_ Pickering double emulsions (PDEs) were prepared by a two-step emulsification method using polyglycerol polyricinoleate (PGPR) and xanthan gum/lysozyme nanoparticles (XG/Ly NPs) as lipophilic and hydrophilic emulsifiers, respectively. The regulation mechanism of the performance of PDEs by XG/Ly NPs was investigated, and the ability of the system to co-encapsulate epigallocatechin gallate (EGCG) and β-carotene was evaluated. The results showed that increasing the XG/Ly NPs concentration can enhance the stability of PDEs. At 60% W_2_ phase percentage and 2.0% XG/Ly NPs, the PDEs showed a smaller droplet size (23.47 ± 2.28 μm) and no phase separation after 21 days of storage. Additionally, the PDEs co-encapsulated system showed higher encapsulation efficiency (EGCG: 89.21%; β-carotene: 99.14%) and maintained high retention of active substances after 8 h of UV illumination (EGCG: 75.51%; β-carotene: 77.24%). As demonstrated by in vitro simulated gastrointestinal digestion assays, the bioaccessibility of EGCG and β-carotene simultaneously encapsulated was improved by 66.0% and 36.2%, respectively, compared with that of individually encapsulated EGCG and β-carotene. Overall, this study provides a new reference for the construction of highly stable PDEs and is promising as a co-encapsulation carrier for environmentally sensitive components.

## 1. Introduction

Double emulsions (DEs), complex multiphase emulsification systems, include water-in-oil-in-water (W/O/W) and oil-in-water-in-oil (O/W/O) types [1]. Compared to O/W/O DEs, W/O/W DEs have a lower oil phase, which is in line with the concept of low-fat diets [2]. Additionally, the unique “sphere-in-sphere” structure of W/O/W DEs makes them ideal carriers for co-encapsulating hydrophilic and lipophilic active ingredients [3,4,5]. Reportedly, Xie et al. prepared W/O/W DEs co-loaded with selenium-enriched peptides and vitamin E by a two-step emulsification method combined with ultrasonication [6]. It was found that the two interfacial layers of the DEs acted as physical barriers and were able to mask the unpleasant flavor of the Se peptide, facilitating its further physiological effects. Although DEs can be employed to encapsulate active ingredients, they are poorly stable. On the one hand, destabilization phenomena such as flocculation, aggregation, and phase separation may occur in DEs as storage time increases [7]. On the other hand, due to the unique three-phase and two-membrane structure, the different osmotic pressures between the inner water phase (W_1_) and the external aqueous phase (W_2_) of DEs can lead to phase migration between the W_1_ and W_2_ phases, which is ultimately manifested in the reduction of stability and leakage of active components of DEs [8]. Therefore, finding new approaches to improve the stability of W/O/W DEs is extremely important.

Pickering particles, a class of amphiphilic solid particles, are used to construct highly stable emulsions due to their excellent interfacial adsorption properties and biocompatibility [9]. In food-grade Pickering emulsion systems, the particle stabilizers need to meet the requirements of food-grade, renewable, and clean labels. Currently, common food-grade particle stabilizers are mainly derived from natural biopolymers (including proteins, polysaccharides, and their complexes) [10]. Protein/polysaccharide complexes constructed based on interactions between natural biopolymers have been shown to exhibit higher interfacial stability, which provides a potential means to enhance the stability of emulsion systems [11]. It was reported that Pickering emulsions prepared by interfacial assembly of zein-xanthan gum complexes or agar-gelatin complexes were effective in suppressing destabilization induced by environmental conditions, such as salt ions, centrifugal force, and heat [12,13].

In recent years, although studies on the preparation of PDEs using Pickering particles as emulsifiers have been reported, there are still some limitations to enhancing the performance of PDEs. For the whole system, PGPR plays a limited role in the initial stabilization of the internal phase, whereas Pickering particles play a crucial role in maintaining the overall stability of PDEs. Pan et al. prepared PDEs using β-cyclodextrin and PGPR as a hydrophilic emulsifier and a lipophilic emulsifier, respectively. At higher concentrations of β-cyclodextrin, although the environmental stability was enhanced, the cost subsequently increased [14]. In addition, PDEs constructed based on gliadin nanoparticles and soy lecithin showed good encapsulation stability, but the phase separation generated during storage limits their further application [15]. These results suggest that single protein-based or polysaccharide-based Pickering particles still have difficulties in constructing highly stable PDEs. In our previous studies, xanthan gum/lysozyme nanoparticles (XG/Ly NPs), a protein-polysaccharide-based complex nanoparticle, were used to stabilize oil-in-water (O/W) Pickering emulsions by virtue of their suitable wettability (contact angle: 78.1°) and small particle size (<100 nm) [16,17]. Specifically, XG/Ly NPs can inhibit destabilization phenomena, such as oil droplet agglomeration and Ostwald ripening in O/W emulsions, by developing a dense interfacial film [18]. Moreover, the interfacial barrier formed by nanoparticles hinders the degradation of lipophilic components encapsulated in the oil phase, facilitating their further physiological activities [19]. Another study found that O/W Pickering emulsions stabilized by XG/Ly NPs showed good pasteurization stability, and β-carotene encapsulated in the emulsions still exhibited high retention after pasteurization at 65 °C for 30 min [20]. Despite their excellent emulsifying ability, XG/Ly NPs lack application in PDEs.

Therefore, we hypothesized that XG/Ly NPs can be used as hydrophilic emulsifiers to construct PDEs with high stability and good encapsulation performance. In this study, PGPR was utilized as a lipophilic emulsifier to stabilize the internal oil-water interface, XG/Ly NPs were utilized as a hydrophilic emulsifier to stabilize the external oil-water interface, and W/O/W PDEs were constructed. The effects of the XG/Ly NPs concentration and W_2_ phase percentage on the physical stability, rheological behavior, and microstructure of the PDEs were investigated. Furthermore, the co-encapsulation of EGCG and β-carotene by PDEs was evaluated by measuring the encapsulation efficiency, photodegradation behavior, and in vitro simulated digestion experiments. This study provides a theoretical basis for the development of highly stable co-encapsulated carriers.

## 2. Materials and Methods

### 2.1. Materials

Lysozyme (Ly, purity > 90%) was supplied by Sinopharm Chemical Reagent Co., Ltd. (Shanghai, China). Xanthan gum (XG) was purchased from Shanghai Yuanye Biotechnology Co., Ltd. (Shanghai, China). Tea oil was kindly supplied by Henan Luda Tea Oil Co., Ltd. (Xinyang, China). PGPR was supplied by Macklin Biochemical Co., Ltd. (Shanghai, China). β-carotene (purity 97%) and EGCG (purity 98%) were sourced from TCI Co., Ltd. (Tokyo, Japan), and Baoji Herbest Biotechnology Co., Ltd. (Baoji, China), respectively.

### 2.2. Preparation of XG/Ly NPs

XG/Ly NPs were prepared using our prior method [16]. Briefly, 0.1% (*w*/*v*) XG and Ly solutions were mixed equally, and the pH of the XG/Ly mixture was adjusted to 11.8 by 1 mol/L NaOH. After heating at 80 °C for 15 min, the mixture was cooled to ambient temperature. Next, after adjusting the pH of the XG/Ly NPs mixture to be neutral by 1 mol/L HCl, it was dialyzed for 24 h. Finally, the XG/Ly NPs were obtained after filtration and lyophilization.

### 2.3. Preparation of Pickering Double Emulsions (PDEs)

**Figure 1:**
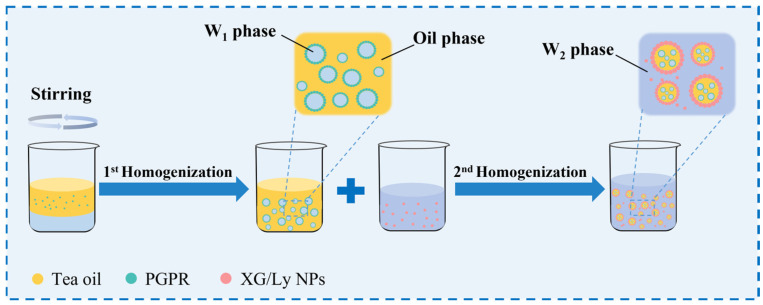
Schematic illustration of the formation mechanism of PDEs stabilized by XG/Ly NPs and PGPR.

PDEs were fabricated by a two-step emulsification method with some modifications [21]. Firstly, 2.0% (*w/w*) sucrose (as an osmotic pressure regulator) was dissolved in deionized water to prepare the W_1_ phase. To obtain the oil phase, 5% (*w*/*w*) PGPR was mixed (55 °C, 100 rpm/min) with tea oil for 0.5 h. The W_2_ phase was prepared by dissolving XG/Ly NPs (1.0%, 1.5%, and 2.0%, *w*/*w*) in deionized water and stirring (25 °C, 200 rpm/min) for 1 h. Subsequently, the W_1_ phase was dropped into the oil phase (water-oil mass ratio of 3:7) and homogenized (18,000 rpm, 3 min) via a homogenizer (Ultra turrax T18, IKA, Staufen, Germany) to obtain the W_1_/O emulsions. Finally, the W_1_/O emulsion was mixed with the W_2_ phase (30%, 40%, 50%, 60%, and 70%, *w*/*w*) and homogenized (12,000 rpm, 2 min) to obtain PDEs. The flowchart for the preparation of PDEs and the formation process of the double emulsion structure are depicted in Figure 1.

EGCG and β-carotene were used as model agents to prepare PDEs that were loaded individually or simultaneously. Specifically, 0.8% (*w*/*w*) EGCG and 0.4% (*w*/*w*) β-carotene were introduced into the W_1_ phase and the oil phase, respectively.

### 2.4. Microstructure of PDEs

The microscopic structure of PDEs was viewed by a confocal laser microscope (SP8, Leica, Wetzlar, Germany). In brief, 1 mL PDEs were dyed with 40 μL Nile Red/Nile Blue A mixed solution (0.1%, *w*/*v*) for 0.5 h. The microstructure of PDEs was observed under a 40× objective microscope. The excitation light sources used were 488 nm (Nile Red) and 633 nm (Nile Blue A). Meanwhile, the overlay CLSM images were saved [22]. In addition, a cryo-scanning electron microscope (Quanta 450, FEI, Hillsboro, OR, USA) was applied to further observe the oil-water distribution of PDEs. Firstly, the PDEs were snap-frozen in liquid nitrogen for 30 s. Subsequently, the microstructures were observed after sublimation and gold plating under high-vacuum conditions. The accelerating voltage and testing temperature were 10 kV and −140 °C, respectively. The magnifications were 4000× and 6000×.

### 2.5. Particle Size and Zeta Potential of PDEs

The particle size of the fresh PDEs was determined by a laser particle sizer (Master Sizer 3000, Malvern, UK). The d_(4,3)_ (volume mean particle size) was applied as the mean particle size of PDEs. The zeta potential of PDEs was measured using a Zetasizer Nano ZS instrument (Malvern Instrument, Malvern, UK) at 25 °C. Prior to testing, the PDEs were diluted 50-fold using deionized water. Each sample was tested at least thrice.

### 2.6. Physical Stability of PDEs

The freshly prepared PDEs were packed in glass vials and stored at 4 °C for 21 days to evaluate their storage stability. The appearance of PDEs was photographed for different storage times, and the microstructure of PDEs after 21 days of storage was observed by CLSM. The creaming index (CI) of PDEs after storage was calculated according to Equation (1).

(1) $$\mathrm{CI}\;(\%)=\frac{H_{s}}{H_{0}}\times 100$$

where H_S_ and H_0_ represent the height of the serum layer and the total height of the PDEs, respectively.

The effects of XG/Ly NPs concentration and W_2_ phase percentage on the centrifugal stability of PDEs were assessed by centrifuging (3000 rpm, 5 min) at 25 °C. Then, the appearance of the PDEs after centrifugation was photographed. Additionally, the thermal stability of PDEs was examined by heat treatment (70 °C, 30 min). After that, the appearance of the PDEs was photographed.

### 2.7. Rheological Behaviors of PDEs

The rheological behaviors of PDEs, including flow behavior, strain sweeps, and frequency sweeps, were characterized by a rotational rheometer (DHR-2, TA, New Castle, DE, USA). A parallel aluminum plate (diameter: 40 mm, gap: 1 mm) was used for the experiments. Firstly, strain sweeps from 0.01% to 100% strain were performed for all PDE samples at a constant frequency of 1 Hz to record the storage (G’) and loss (G″) moduli. Additionally, a linear viscoelastic zone (LVZ) was identified. Then, the viscosity of the samples was recorded from 0.1 s^−1^ to 100 s^−1^. At 0.1% strain (within the LVZ), frequency scans from 0.1 rad/s to 100 rad/s were carried out on the samples. The temperature for all the above rheological measurements was 25 °C.

### 2.8. Encapsulation Efficiency (EE) and Loading Efficiency (LE) of PDEs

The EE of EGCG was assayed based on a previous report with some modifications [23]. Briefly, 0.5 mL of PDEs were mixed with 1.5 mL of deionized water, and after vertexing for 30 s and centrifugation (13,000 rpm, 25 min), the aqueous phase was collected with a syringe. Next, 0.5 mL of the aqueous phase was mixed with 1 mL of Folin-Ciocalteau reagent for 5 min, followed by the addition of 2 mL of 10% (*w*/*v*) Na_2_CO_3_ solution, shaking well, and the reaction was carried out in a dark room for 0.5 h. The reaction solution was filtered using a 0.45 μm filter, and the absorbance of the filtrate at 765 nm was determined by a UV/Vis spectrophotometer (Lambda 465, PerkinElmer, Waltham, MA, USA). The unencapsulated EGCG content was calculated using an EGCG standard curve.

The EE of β-carotene was determined based on a previous approach, with some changes [24]. First, 1 mL of PDEs was vortexed with 3 mL of n-hexane for 30 s and then centrifuged (5000 rpm, 5 min) to collect the supernatant. Next, the supernatant was then diluted to a suitable multiple, and its absorbance at 450 nm was measured by a UV-visible spectrophotometer (Lambda 465, PerkinElmer, Waltham, MA, USA). The content of unencapsulated β-carotene was calculated by a standard curve for β-carotene. The EE and LE of EGCG and β-carotene were calculated according to the following equations [25]:

(2) $$\mathrm{EE}\;\left(\%\right)=\frac{A_{0}-A_{w}}{A_{0}}\times 100$$

(3) $$\mathrm{LE}\;\left(\%\right)=\frac{A_{0\;}-{\;A}_{w}}{A_{p}}\times 100$$

wherein A_0_ represents the initial total amount of EGCG or β-carotene added in the W_1_ phase or the oil phase, A_w_ represents the unencapsulated EGCG or β-carotene content, and A_p_ represents the total amount of EGCG or β-carotene in the W_1_ phase or the oil phase.

### 2.9. Photostability of EGCG and β-Carotene

Free EGCG, free β-carotene, and PDEs encapsulating EGCG/β-carotene were subjected to UV irradiation, and the retention of EGCG and β-carotene was determined at different irradiation times. The emulsion samples (1 g) were diluted with water, and 10 mL n-hexane was added and vortexed for 30 s to extract the β-carotene. The extraction was repeated twice, and then the extracts were combined. Then, the absorbance of the extract at 450 nm was determined [26]. Additionally, the lower aqueous phase was aspirated with a syringe, and the content of EGCG was assayed with reference to the procedure described in Section 2.8. The retention of EGCG and β-carotene was calculated using Equation (4).

(4) $$\mathrm{Retention}\;\mathrm{rate}\;(\%)=\frac{C_{t}}{C_{0}}\times 100$$

where C_0_ denotes the content of EGCG or β-carotene without UV irradiation, and C_t_ denotes the content of EGCG or β-carotene after UV irradiation for t h.

The photodegradation behavior of EGCG and β-carotene was further explored by the degradation rate constant (k_1_) and half-life (t_1/2_), which were calculated by the following equations:

(5) $$\ln\left(\frac{C_{t}}{C_{0}}\right)=-{\;k}_{1}t$$

(6) $${\;t}_{1/2}=\frac{\ln2}{k_{1}}$$

### 2.10. Digestion Behavior of PDEs

The simulated gastric fluid (SGF) and simulated intestinal fluid (SIF) were prepared following a previous method with some modifications [18]. For simulated gastric digestion, 9 mL of fresh PDEs was mixed with 9 mL of SGF and blended at 37 °C for 120 min. Subsequently, the pH of the gastric digestion mixture was rapidly adjusted to 7.0, and 18 mL of SIF was added at 37 °C for 120 min. In addition, the pH was maintained at 7.0 by titrating the NaOH solution to assess the release level of free fatty acids (FFA) from PDE samples. NaOH consumption during simulated intestinal digestion was recorded, and the FFA release level was calculated using Equation (7).

(7) $$\mathrm{FFA}\;\mathrm{release}\;(\%)\;=\frac{V_{N}\times C_{N}\times M_{L}}{2W_{L}}\times 100$$

where V_N_ indicates the consumption of NaOH (L) and C_N_ indicates the concentration of NaOH (1 mol/L). M_L_ and W_L_ indicate the total weight (g) and molecular weight (g/mol) of tea oil, respectively.

Furthermore, the FFA release during simulated intestinal digestion of PDEs was evaluated by a pseudo-first-order kinetic model and calculated by Equation (8) [27].

(8) $$\ln(\frac{\varphi_{m}-\varphi_{t}}{\varphi_{m}})=-{\;k}_{2}t$$

where φ_m_ denotes the total FFA release level, φ_t_ denotes the FFA release level at time t, and k_2_ denotes the release rate constant (min^−1^).

To determine the bioaccessibility of EGCG and β-carotene, the mixture after simulated intestinal digestion was centrifuged (8000 rpm,15 min) at 4 °C to extract the micelles. Then, the contents of EGCG and β-carotene in the micelles were measured with reference to the method described in Section 2.9. The bioaccessibility was calculated based on the following equation:

(9) $$\mathrm{Bioaccessibility}\;\left(\%\right)=\frac{C_{1}}{C_{0}}\times 100$$

where C_1_ represents the content of EGCG or β-carotene in the micelles, and C_0_ represents the initial content of EGCG or β-carotene in the PDEs.

### 2.11. Statistical Analysis

All experiments were performed at least three times, and the obtained data are reported as mean ± standard deviation. One-way ANOVA and Duncan’s multiple comparison tests were applied using SPSS 25.0 software (SPSS Inc., Chicago, IL, USA). When *p* < 0.05, the data were considered significantly different.

## 3. Results and Discussion

### 3.1. Microstructure, Particle Size, and Zeta Potential

**Figure 2:**
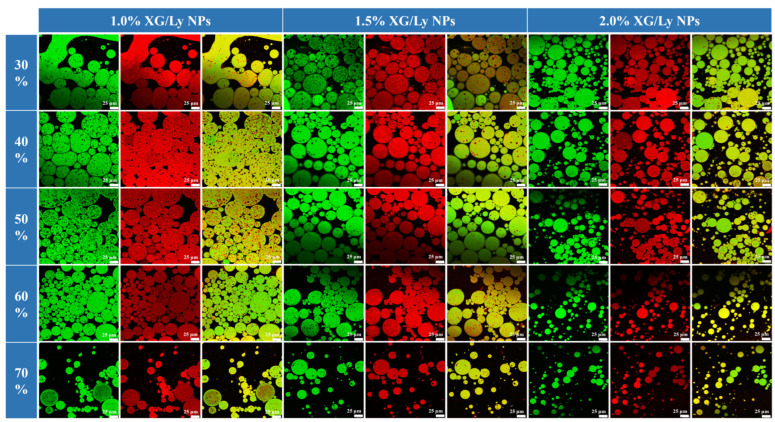
The CLSM images of PDEs with different W_2_ phase percentages (30–70%) and XG/Ly NPs concentrations (1.0–2.0%, *w*/*w*).

To verify the successful preparation of the W/O/W PDEs, the microstructure of the emulsion samples was visualized using CLSM. As shown in Figure 2, strong green fluorescence was observed in the oil phase stained with Nile Red. The black areas inside and outside the oil phase denote the unstained aqueous phase. As particle stabilizers, XG/Ly NPs were distributed in the oil-water interfacial layer, which was stained by Nile Blue A and showed red fluorescence, which was different from O/W emulsions. This finding was consistent with previous reports [28,29]. Therefore, in this study, W/O/W PDEs with a three-phase and two-compartment structure were successfully prepared.

**Figure 3:**
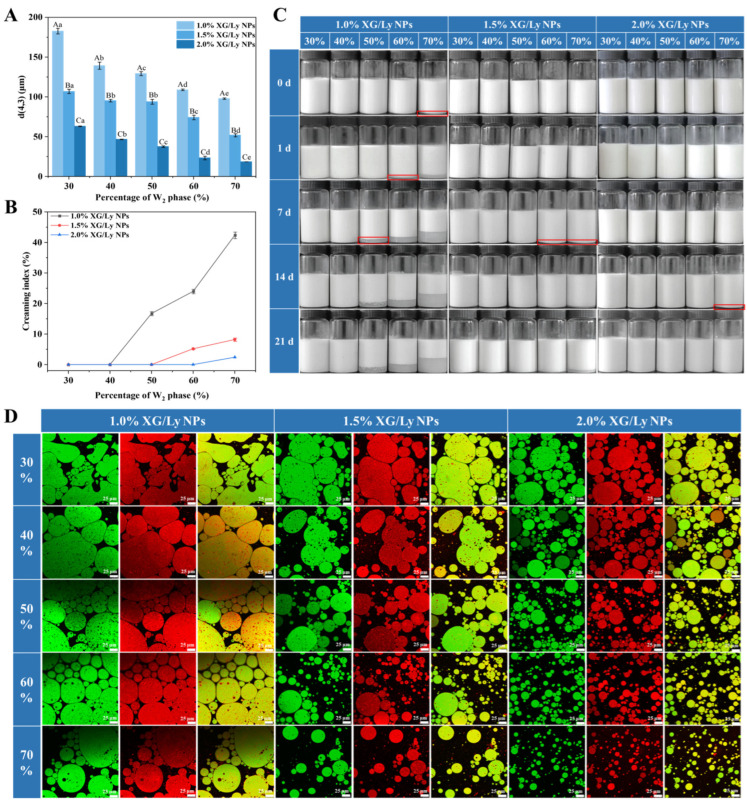
The average particle size (**A**) of fresh PDEs at different W_2_ phase percentages (30–70%) and XG/Ly NPs concentrations (1.0–2.0%, *w*/*w*), as well as the creaming index (**B**), appearance (**C**), and the CLSM images (**D**) after 21 days of storage. Different uppercase/lowercase letters denote significant differences (*p* < 0.05). Note: The red wireframe in (**C**) indicates the markers where the serum layer first appeared in the samples.

Further analysis revealed that varying the W_2_ phase percentage and the concentration of XG/Ly NPs could effectively regulate the droplet size of PDEs (Figure 2). At 30% W_2_ phase percentage, there were more irregularly shaped oil droplets in the PDEs with 1.0% XG/Ly NPs. It was attributed to the fact that too few XG/Ly NPs at high oil phase percentages resulted in a thin interfacial layer, which could easily polymerize and form larger oil droplets upon collision [30]. However, as the XG/Ly NPs concentration increased, more XG/Ly NPs were adsorbed in the oil-water interfacial layer, which provided a strong physical barrier to separate the oil droplets and effectively inhibited droplet aggregation, thus maintaining PDEs with small droplet sizes (Figure 2). For example, at 60% W_2_ phase percentage, compared with 1.0% XG/Ly NPs, the droplet size of PDEs stabilized by 2.0% XG/Ly NPs was significantly reduced from 109 ± 1 μm to 23.47 ± 2.28 μm (*p* < 0.05) (Figure 3A). Moreover, the droplet size was smaller than that of PDEs stabilized with the same concentration of phycocyanin or beet pectin-bovine serum albumin nanoparticles [31,32].

As seen in Figure S1, increasing the concentration of XG/Ly NPs reduced the polydispersity index (PDI) of PDEs, indicating an increase in the droplet homogeneity of PDEs, which was in line with the results of CLSM images. Additionally, the zeta potentials of PDEs at different concentrations of XG/Ly NPs are shown in Table S1. Generally, when the absolute value of the zeta potential exceeds 30 mV, the electrostatic repulsion within the emulsion system effectively hinders the flocculation of droplets, thus exhibiting good stability [33]. Reportedly, the zeta potential of XG/Ly NPs was lower than −30 mV, indicating good electrostatic stability [17]. In the present study, with the increase of XG/Ly NPs concentration, the absolute value of zeta potential of PDEs increased, especially at 2.0% XG/Ly NPs, where the value of zeta potential reached −31.1 ± 0.36 mV. This indicated that there was sufficient electrostatic repulsive force in PDEs to stabilize the system.

### 3.2. Physical Stability Analysis

The appearance and CI of PDEs during storage are shown in Figure 3B,C. For freshly prepared PDEs, only PDEs stabilized by 1.0% XG/Ly NPs at a 70% W_2_ phase percentage appeared as a serum layer. When the W_2_ phase percentage was lower than 40%, the higher oil phase content increased the droplet stacking density and limited the droplet movement, which was an important reason for the absence of a serum layer in the PDEs after storage [34]. At 1.0% XG/Ly NPs, all PDEs with more than 40% W_2_ phase showed a serum layer, and the height increased as the W_2_ phase percentage increased (Figure 3B). Notably, an increase in the particle concentration delayed the appearance of the serum layer during storage. It was because increasing the particle concentration could promote more XG/Ly NPs to adsorb on the oil-water interfacial layer, forming a complete physical barrier to prevent the aggregation of oil droplets [18]. Additionally, as seen in Figure 3D, the PDEs stabilized by 2.0% XG/Ly NPs retained small droplet sizes after 21 days of storage at 60% W_2_ phase percentage and exhibited good storage stability. Previous reports have shown that when stabilizing PDEs with wheat gliadin nanoparticles, a particle concentration of 5% is required to keep the PDEs free of the serum layer for 14 days [5]. In contrast, the XG/Ly NPs were able to show superior stabilization at lower concentrations and lower stabilizer dosages.

**Figure 4:**
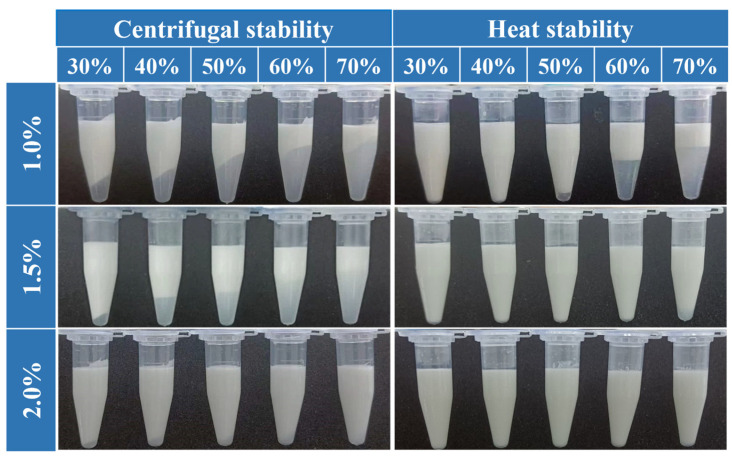
The centrifugal stability and heat stability of PDEs at different W_2_ phase percentages (30–70%) and XG/Ly NPs concentrations (1.0–2.0%, *w*/*w*).

High-speed centrifugation is a convenient approach for assessing the physical stability of PDEs [35]. After centrifugation, for PDEs stabilized by 1.0% and 1.5% XG/Ly NPs, phase separation was observed for the samples at different W_2_ phase percentages (Figure 4). However, the phase separation was suppressed with the increasing concentration of XG/Ly NPs. It was probably because the W_2_ phase viscosity increased from 1.57 Pa·s to 26.05 Pa·s when the particle concentration was increased from 1.0% to 2.0%, which restrained the centrifugation-induced droplet aggregation (Figure S2). Meanwhile, the particle network formed by the XG/Ly NPs between the droplets increased the structural stability of the PDEs, which could effectively resist the destabilizing behavior induced by the externally applied centrifugal force (Figure 4) [18]. The enhanced centrifugal stability was also noted in Pickering emulsions stabilized with higher concentrations of tea residue protein/xanthan gum particles [30].

The heat stability of PDEs was determined by heat treatment at 70 °C for 30 min (Figure 4). At lower XG/Ly NPs concentrations, the heat treatment caused delamination of the PDEs. As the concentrations of nanoparticles increased, the intact interfacial adsorbent layer conferred better dispersion properties to the droplets, which suppressed heating-induced droplet aggregation [36]. It was consistent with the results of centrifugal stability.

### 3.3. Rheological Behaviors Analysis

**Figure 5:**
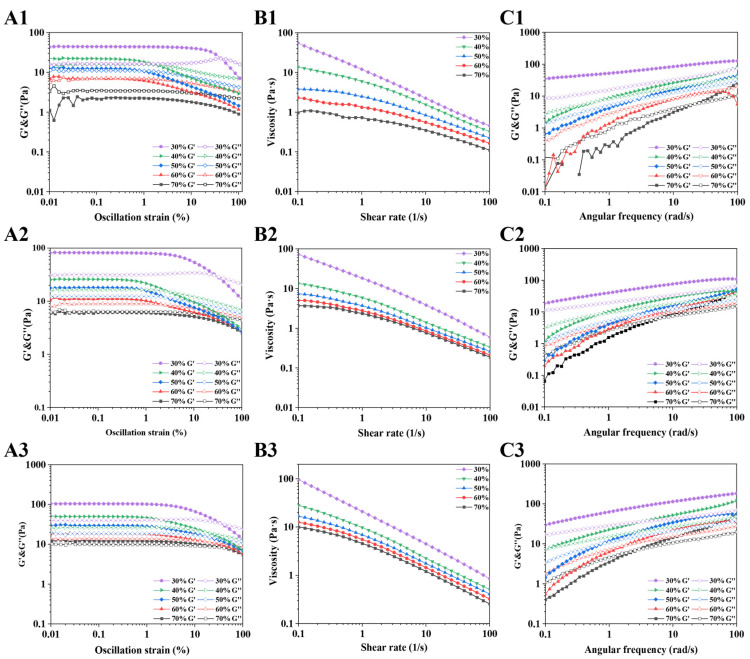
The oscillatory strain sweep (**A1**–**A3**), steady state shear sweep (**B1**–**B3**), and frequency sweep (**C1**–**C3**) curves of PDEs at different W_2_ phase percentages (30–70%) and XG/Ly NPs concentrations (1:1.0%, 2:1.5%, 3:2.0%).

Firstly, strain sweeps were performed for PDEs (Figure 5A1–A3). The PDE samples showed good resistance to deformation, as G’ and G″ remained constant within a specific range of strain (i.e., LVZ). Based on the LVZ of different samples, 0.1% strain was selected for subsequent rheological tests.

As shown in Figure 5B1–B3, the viscosity of all samples reduced gradually as the shear rate increased and showed the characteristics of shear thinning [37]. The viscosities of the PDEs decreased when the W_2_ phase percentage increased. Previous research has shown that the viscosity of emulsions increases with an increase in oil content. At a lower W_2_ phase percentage, PDEs have a higher oil content, tightly packed oil droplets, and reduced fluidity, which contribute to their increased viscosity. At the same W_2_ phase percentage, the viscosity improved as the concentration of XG/Ly NPs increased. It was ascribed to the higher adsorption of XG/Ly NPs in the interfacial layer at high XG/Ly NPs concentrations, which increased the interactions between the oil droplets [38].

The frequency scanning curves of the PDEs are shown in Figure 5C1–C3. Both G’ and G″ of the PDEs increased with increasing frequency. The higher W_2_ phase percentage implied a lower oil phase percentage, resulting in a weaker viscoelasticity of the emulsions. This change was more obvious in PDEs stabilized by low XG/Ly NPs concentrations at high W_2_ phase percentages. In other words, PDEs mainly showed viscous characteristics at low frequencies and elastic characteristics at high frequencies [39]. However, increasing the concentration of XG/Ly NPs can improve the viscoelasticity of PDEs for a more stable interfacial structure, which is manifested as a shift in the viscoelastic transition point from high to low frequency. A better improvement was observed in the sample stabilized with 2.0% XG/Ly NPs.

Based on these results, in order to construct a PDE system with low oil content and high stability, we further explored the effects of XG/Ly NPs concentrations on the different properties of PDEs in the 60% W_2_ phase.

### 3.4. Cryo-SEM Observations

**Figure 6:**
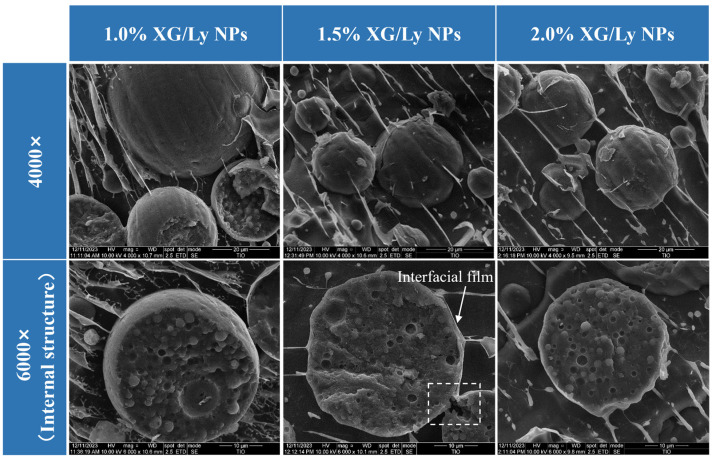
The Cryo-SEM images of PDEs stabilized with different XG/Ly NPs concentrations (1.0–2.0%, *w*/*w*) at 60% W_2_ phase percentage.

Cryo-SEM is one of the powerful tools for observing the different phases inside PDEs and their interfacial structures [28]. Typical spherical oil droplets are shown in Figure 6. In addition, at 1.0% XG/Ly NPs, the PDEs showed some large oil droplets in the Cryo-SEM images. With the increase in XG/Ly NPs concentration, the oil droplet size decreased, which was consistent with the results of particle size (Figure 3A). Notably, when the concentration of XG/Ly NPs was lower than 2.0%, although the Cryo-SEM images showed a particle network, agglomerating oil droplets were also clearly observed. It was likely due to the weak electrostatic repulsion provided by the low concentrations of XG/Ly NPs to the PDEs that the oil droplets were prone to collisional agglomeration, resulting in poorer stability of the PDEs (Table S1). In contrast, with the addition of 2.0% XG/Ly NPs, the interfacial film on the surface of the oil droplets was denser, and a stable particle network was formed between the oil droplets, which effectively enhanced the structural stability of the whole emulsion system. It was also the reason that the PDEs stabilized by 2.0% XG/Ly NPs had higher physical stability.

To further confirm the existence of a three-phase structure in the PDEs, Cryo-SEM cross-sectional images of the PDEs were captured (Figure 6). Some pits were visible within the oil droplets, which were attributed to the sublimation of water in the W_1_ phase under high-vacuum conditions. This finding provides additional evidence for the formation of W/O/W PDEs, which is consistent with the findings observed in the CLSM images (Figure 2). Additionally, a similar cross-sectional structure was observed in a W/O/W double emulsion stabilized by a whey protein concentrate-pectin complex and PGPR [40].

### 3.5. Encapsulation of EGCG and β-Carotene

**Figure 7:**
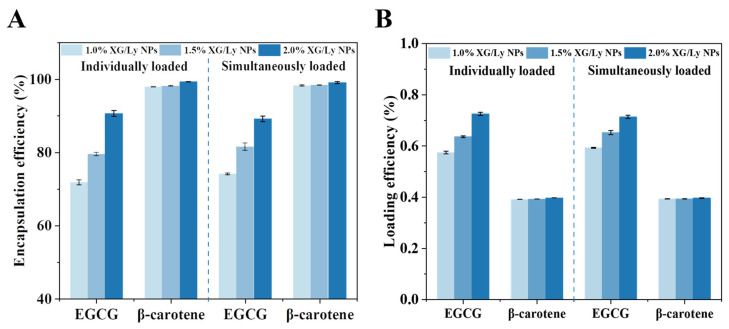
The encapsulation efficiency (**A**) and loading efficiency (**B**) of EGCG and β-carotene in PDEs.

Due to the unique multiphase structure, PDEs are favorable encapsulation carriers for hydrophilic and hydrophobic actives. The effect of particle concentration on the encapsulation ability of PDEs was evaluated using EGCG and β-carotene as model substances. Generally, the EE of EGCG and β-carotene in PDEs increased with increasing XG/Ly NPs concentration (Figure 7A). For 1.0% XG/Ly NPs, the EE of EGCG and β-carotene was 71.80% and 97.91%, respectively. Whether individually or simultaneously loaded, the EEs were similar in the two model substances. An analogous result was found in a double emulsion system with simultaneously loaded curcumin and catechin [41]. At 2.0% XG/Ly NPs, the improved integrity of the oil-water interfacial layer led to the suppression of interpenetration between the W_1_ and W_2_ phases, which conferred a higher EE to the PDEs. In the simultaneous encapsulation group, the EEs of EGCG and β-carotene were 89.21% and 99.14%, respectively. The highly satisfactory EEs demonstrate the potential of PDEs for the encapsulation of hydrophilic and hydrophobic substances and set the stage for their application. In addition, the LE and EE had similar trends with the increase in particle concentration (Figure 7B). For the co-loaded group, the LE of EGCG and β-carotene in PDEs with 2.0% XG/Ly NPs were 0.71% and 0.40%, respectively. It was superior to other reports of double emulsions loaded with active ingredients, such as grape seed proanthocyanidin (LE = 0.15%) and quercetin (LE = 0.097%) [5,25].

### 3.6. Photostability Analysis of PDEs

**Figure 8:**
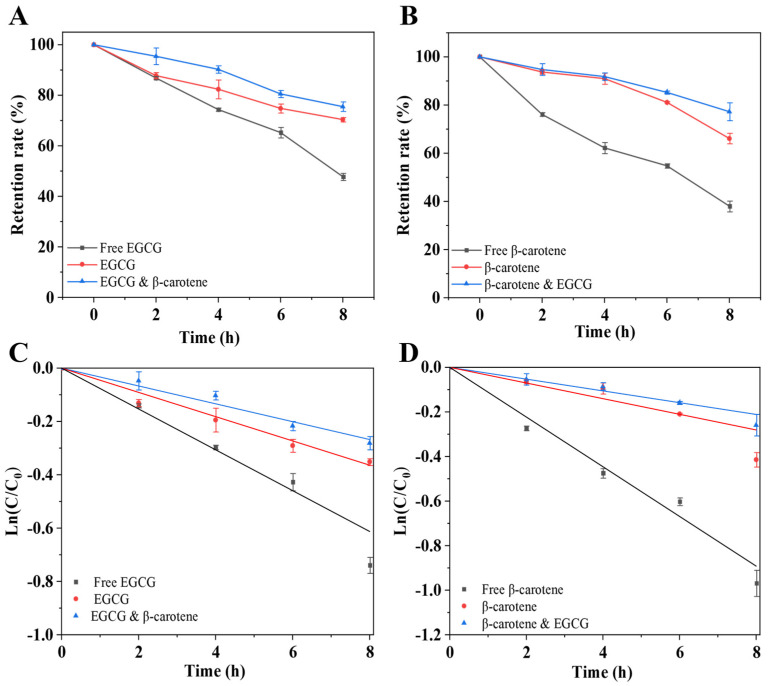
The photostability ((**A**): EGCG, (**B**): β-carotene) and half-life function ((**C**): EGCG; (**D**): β-carotene) of EGCG and β-carotene under different load conditions.

To evaluate the protective effects of PDEs on EGCG and β-carotene, UV irradiation experiments were performed on PDEs under different loading conditions. The retention rates of EGCG and β-carotene decreased continuously with the increase in light irradiation time (Figure 8A,B). After 8 h of UV irradiation, the retention of free EGCG and β-carotene decreased to 47.79% and 38.04%, respectively, which was significantly lower than the retention of EGCG and β-carotene in the PDEs. It was because the closely arranged XG/Ly NPs in the oil-water interfacial layer blocked the photodegradation of EGCG and β-carotene by UV light [18]. Reportedly, PDEs co-encapsulating vitamin D and curcumin also show enhanced UV-resistant properties [42]. Compared with individual loading, the retention of EGCG increased from 70.38% to 75.51%, and that of β-carotene increased from 66.12% to 77.24% in PDEs under co-loading. This result indicated that EGCG and β-carotene exhibited a synergistic effect when co-loaded, which can further delay the photodegradation process. Similar findings were obtained for the O/W/O double emulsion system [43].

To further analyze the photodegradation behaviors of EGCG and β-carotene, the retention rate data were fitted into first-order kinetic equations (Figure 8C,D). From the results, all groups of data were well fitted (R^2^ > 0.98) (Table 1). The half-lives of free EGCG and β-carotene were 9.05 h and 6.22 h, respectively. Under co-encapsulation, the half-lives of EGCG and β-carotene were higher than those of the free groups. These results illustrated that PDEs stabilized with XG/Ly NPs and PGPR can be used to construct highly stable double emulsion co-encapsulation systems.

### 3.7. In Vitro Simulated Digestion

**Figure 9:**
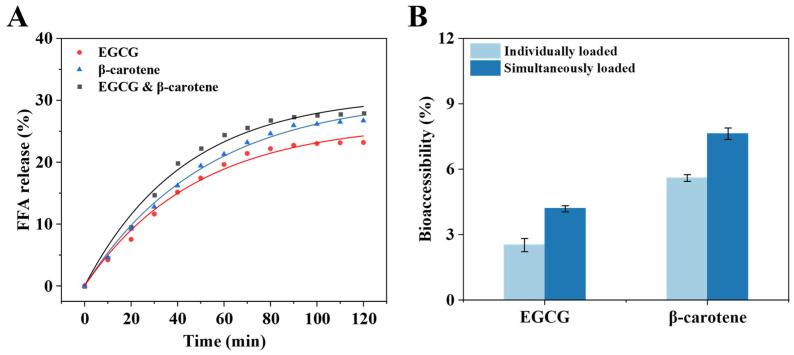
FFA release levels (**A**) and bioaccessibility (**B**) of EGCG and β-carotene in PDEs after intestinal digestion.

The in vitro digestive properties of emulsions are critical for the release and absorption of active ingredients. The degree of lipolysis of PDEs was assessed based on the FFA release level during simulated intestinal digestion. Meanwhile, in order to analyze the simulated intestinal digestion process, a pseudo-first-order kinetic model was applied to fit the FFA release curves, and a favorable fit was obtained (R^2^ > 0.98) (Table 2). As shown in Figure 9A, due to the action of lipase and bile salts, the FFA release level of the samples in each group increased rapidly for about the first 40 min, followed by a slow progression, similar to the results reported previously [25]. Compared to the individually loaded group, the co-loaded group showed a higher level of FFA release (27.94%), corresponding to a greater release rate constant (Table 2). Overall, the FFA release level in the PDEs system was below 30%, regardless of the encapsulation method. It may be due to the interfacial membrane formed by the XG/Ly NPs hindering the interaction between lipase, bile salts, and oil droplets, thus reducing the decomposition of oil in PDEs [44].

Figure 9B shows the bioaccessibility of EGCG and β-carotene in the PDEs after simulated intestinal digestion. The bioaccessibility of both was affected by the loading mode. During the intestinal digestion period, the action of lipase on the oil phase resulted in the gradual collapse of the double emulsion structure and the release of EGCG from the W_1_ phase. When loaded individually, EGCG showed low bioaccessibility (2.5%). It was associated with the extreme instability of EGCG in neutral intestinal digestive fluids [41,45]. Notably, the bioaccessibility of EGCG was enhanced by 66.0% when it was co-loaded. A previous report showed a similar protective effect in oil-in-water emulsion gels co-loaded with EGCG and β-carotene [46]. At different loading modes, β-carotene exhibited similar changes in bioaccessibility to EGCG. The bioaccessibility of β-carotene was elevated by about 36.2% under co-loading compared to when loaded individually. This change may be due to the following reasons. Firstly, during intestinal digestion, the micellar phase formed by hydrolysis of the oil phase solubilized and protected the fat-soluble β-carotene [5]. When loaded simultaneously, PDEs showed a higher FFA release level, indicating a greater degree of lipid hydrolysis, which facilitated the formation of more mixed micelles to protect β-carotene [45]. Secondly, due to incomplete encapsulation, EGCG was distributed in the W_1_ and W_2_ phases, while β-carotene was distributed in the oil phase. During in vitro simulated digestion, the W_2_ phase with EGCG first contacted and mixed with simulated gastric/intestinal fluids, which inhibited the degradation of β-carotene in the oil phase and provided more possibilities for its further absorption and utilization.

## 4. Conclusions

This study successfully constructed high-stability W/O/W PDEs using XG/Ly NPs as hydrophilic stabilizers and PGPR as lipophilic emulsifiers, and achieved the co-encapsulation of EGCG and β-carotene. At a 60% W_2_ phase percentage, the droplet size of the PDEs decreased with an increase in the concentration of XG/Ly NPs, while the viscoelasticity and physical stability were enhanced. The PDEs stabilized by 2.0% XG/Ly NPs had a more stable interfacial layer and showed a higher encapsulation efficiency (EGCG: 89.21%; β-carotene: 99.14%). UV irradiation experiments showed that PDEs could serve as potential protective carriers to effectively inhibit the photodegradation behavior of EGCG and β-carotene. Under co-encapsulation, the retention of EGCG and β-carotene was greater than 70% after 8 h of UV irradiation. Additionally, based on the results of in vitro gastrointestinal digestion assays, EGCG and β-carotene in PDEs showed higher bioaccessibility under co-encapsulation. These findings provide a potential strategy for the co-encapsulation of hydrophilic and lipophilic active ingredients in PDE systems.

## Figures and Tables

**Table 1:** Degradation rate constants (k_1_), half-lives (t_1/2_), and regression coefficients (R^2^) for the first-order kinetic models of EGCG and β-carotene under different encapsulation conditions.

Samples	Rate Constant (k_1_)	t_1/2_ (h)	R^2^
Free EGCG	0.0767	9.05	0.990
EGCG	0.0455	15.23	0.990
EGCG and β-carotene	0.0335	20.71	0.985
Free β-carotene	0.111	6.22	0.984
β-carotene	0.0351	19.74	0.994
β-carotene and EGCG	0.0264	26.25	0.995

**Table 2:** Degradation rate constants (k_2_), half-lives (t_1/2_), and regression coefficients (R^2^) for the first-order kinetic models of EGCG and β-carotene under different encapsulation conditions.

Samples	k_2_ (min^−1^)	R^2^
EGCG	0.0209	0.990
β-carotene	0.0192	0.996
EGCG and β-carotene	0.0233	0.987

## Data Availability

The original contributions presented in the study are included in the article/Supplementary Materials, and further inquiries can be directed to the corresponding authors.

## Supplementary Materials

The following supporting information can be downloaded at: https://www.mdpi.com/article/10.3390/foods14010098/s1, Figure S1 Polydispersity index (PDI) of PDEs at different W_2_ phase percentages and XG/Ly NPs concentrations. Figure S2 W_2_ phase viscosity of PDEs. Note: The W_2_ phase is XG/Ly NPs solution. Table S1 Zeta potentials of PDEs stabilized by different concentrations of XG/Ly NPs at 60% W_2_ phase percentage.
