# Supplementary material for: Construction of Pickering Double Emulsions Based on Xanthan Gum/Lysozyme Nanoparticles: Structure, Stability, and Co-Encapsulation of Epigallocatechin Gallate and β-Carotene
Source: Foods. 2025 Jan 2;14(1):98. doi: 10.3390/foods14010098 (PMC11719661; doi:10.3390/foods14010098)
Supplement: Supplementary file 1 [file foods-14-00098-s001.zip › foods-3344836-supplementary.pdf]

Supplementary Materials

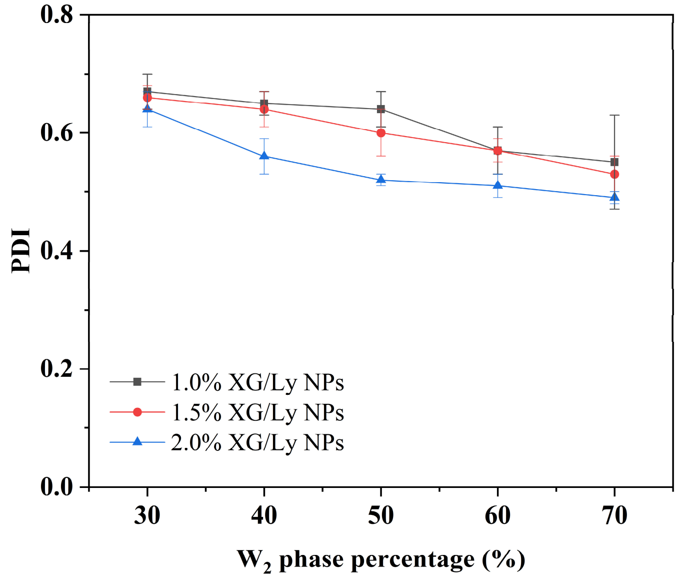

Figure S1 Polydispersity index (PDI) of PDEs at different W_2 phase percentages and XG/Ly NPs concentrations

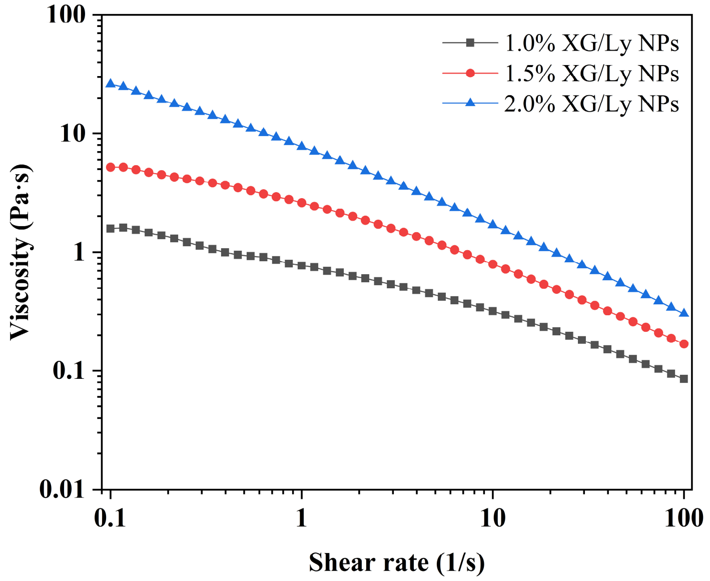

Figure S2 W_2 phase viscosity of PDEs. Note: The W_2 phase is XG/Ly NPs solution.

Table S1 Zeta potentials of PDEs stabilized by different concentrations of XG/Ly NPs at 60% W_2 phase percentage.

XG/Ly NPs concentration (%)	Zeta potential（mV)
1.0	-28.00±0.35[b]
1.5	-28.83±0.75[b]
2.0	-31.10±0.36[a]

Note: Different lowercase letters denote significant differences ($P < 0.05$).
